# Early Detection of Atrophy of Foot Muscles in Chinese Patients of Type 2 Diabetes Mellitus by High-Frequency Ultrasonography

**DOI:** 10.1155/2014/927069

**Published:** 2014-08-06

**Authors:** Xiaohui Wang, Liang Chen, Weiwei Liu, Benli Su, Yuhong Zhang

**Affiliations:** ^1^Department of Ultrasound, Huiqiao VIP and International Medical Centre, Nanfang Hospital, Southern Medical University, Guangzhou 510515, China; ^2^Department of Neurobiology, School of Basic Medical Sciences, Southern Medical University, Guangzhou 510515, China; ^3^Library of Dalian Medical University, Dalian 116044, China; ^4^Department of Endocrinology, Second Affiliated Hospital of Dalian Medical University, Dalian 116023, China; ^5^Department of Diagnostic Ultrasound, Second Affiliated Hospital of Dalian Medical University, 467 Zhongshan Road, Dalian 116023, China

## Abstract

The aim of this study was to evaluate the diagnostic value of high-frequency ultrasonography in detecting atrophy of foot muscles in Chinese patients of type 2 diabetes mellitus (T2DM). Chinese patients of T2DM with (*n* = 56) or without (*n* = 50) diabetic peripheral neuropathy (DPN) and the control subjects (*n* = 50) were enrolled. The nondominant foot of all subjects was examined with high-frequency ultrasonography. The transverse diameter, thickness, and cross-sectional area of the extensor digitorum brevis muscle (EDB) and the thickness of the muscles of the first interstitium (MILs) were measured. The results showed that the ultrasonographic transverse diameter, thickness, and cross-sectional area of EDB and the thickness of MILs in patients of T2DM with DPN were significantly smaller than those in patients of T2DM without DPN (all *P* < 0.01) and those in the control subjects (all *P* < 0.01). The transverse diameter and cross-sectional area of the EDB and thickness of MILs in patients of T2DM without DPN were significantly smaller than those of the control subjects (all *P* < 0.01). In conclusion, the atrophy of foot muscle in Chinese T2DM patients can be detected by high-frequency ultrasonography. Notably, ultrasonography may detect early atrophy of foot muscles in patients without DPN.

## 1. Introduction

Diabetes mellitus is a major health problem which affects approximately 170 million people worldwide in 2001 and estimated more in the future [1, 2]. Importantly, developing countries with rapid change of life style, such as China, are facing the problem as well [3]. The population of type 2 diabetes mellitus (T2DM) especially increases dramatically in the Chinese population [4–6]. Diabetic neuropathy is a common and chronic complication of diabetes, which is responsible for most limb amputations and morbidity partly because of foot muscles atrophy [7, 8]. Notably, the extent of foot muscles atrophy is also a reliable measurement of the neuropathic process [9]. Thus, it is important to determine the degree of foot muscles atrophy to help evaluate the neuropathic process and decide the corresponding strategies of timely therapy [10, 11].

Foot muscles atrophy in the Danish diabetic patients of both type 1 and type 2 with diabetic peripheral neuropathy (DPN) has been detected with ultrasonography [12, 13]. As far as we know, the method has not been tested in Chinese diabetic patients of T2DM by now. Additionally, the sensitivity of the detection needs to be tested in the Chinese since the muscle size of them is smaller than that of the Caucasians such as the Danes [14]. The present study explored whether ultrasonography can detect the foot muscles atrophy in Chinese diabetic patients of T2DM including those before and after the concurrency of DPN.

## 2. Materials and Methods

### 2.1. Participants

One hundred and six Chinese inpatients of T2DM (56 males, 50 females, mean age of 61 ± 6 years, range of 38–78 years) were recruited from the Department of Endocrinology of the Second Affiliated Hospital of Dalian Medical University. All patients were diagnosed by experienced physicians in accordance with the World Health Organization guidelines of diagnosis and classification [15]. Patients were then divided into two groups according to the diagnosis of DPN. The criteria of DPN are referring to the principles of the San Antonio Consensus criteria [16]. Neuropathy symptom score and neuropathy disability score were evaluated [17, 18]. In the group of patients with DPN, there were fifty-six patients (30 males, 26 females, mean age of 63 ± 7 years, range of 44–77 years); in the group of patients without DPN, there were fifty patients (26 males, 24 females, mean age of 59 ± 10 years, range of 38–78 years). Fifty matched control subjects (25 males, 25 females, mean age of 59 ± 7 years, range of 49–78 years) were enrolled among the hospital staff. All subjects gave informed consent to the study which was approved by the local ethics committee.

All subjects were able to walk by themselves without a history of foot surgery or arterial insufficiency of the lower extremities. Subjects with open lesions on their feet, peripheral vascular disease (symptoms of claudication and absence of peripheral pulses, ankle brachial index [ABI] < 0.7) [19], severe cardiac or lung disease, cancer, acute or chronic musculoskeletal disease, other neurological diseases, other endocrine disorders, or symptomatic peripheral artery disease and those who were carrying out motor function rehabilitation were excluded. Sports professionals were excluded.

### 2.2. Data Collection

Both vibration perception threshold (VPT) by biothesiometry and pressure perception using Semmes-Weinstein monofilaments (SWM) have been used to measure the extent of neuropathy [20]. Age, gender, BMI, diabetic duration, HbA1C, and ankle brachial index were recorded and obtained.

### 2.3. Ultrasonographic Examination

All ultrasonographic examinations were performed by the same examiner using a scanner with a linear array real-time high-frequency ultrasonic probe (GE LOGIQ E9, 9–15 MHz). The subjects were placed in a supine position with the nondominant foot placed on a plastic ramp to keep the ankle joint in a neutral position, in case of the adverse effect of possibly excessive extension or contraction. The examiner detected two groups of muscles, including the extensor digitorum brevis muscle (EDB) and the muscles between the first and second metatarsal bone (MILs). The transverse diameter, thickness, and cross-sectional area of the EDB (with the 15 MHz beam) and thickness of the MILs (with the 9 MHz beam) were detected. The examination methods of either muscle group were implemented as described before [13]. The Patients with obvious foot muscles atrophy were asked to perform voluntary contraction of muscles to help define the muscle before examination. The ultrasonograms were downloaded to computers and each parameter was measured five times by the included measurement and analysis package (GE LOGIQ E9). After exclusion of the extreme values, the average of the remaining values was used for further analysis.

### 2.4. Statistical Analysis

All data was analyzed with professional software of statistics (SPSS version 13.0, SPSS Inc., Chicago, USA). The quantitative data was represented as mean and standard deviation ($\overset{-}{x}\pm \text{SD}$). The variable coefficients (CV) of values of each parameter in control subjects were calculated with the formula $\text{CV}=\text{SD}/\overset{-}{x}\times 100\%$. Multiple mean comparison was analyzed by the one-way analysis of variance (ANOVA) followed by post hoc LSD test. *P* < 0.05 was considered statistically significant.

## 3. Results

The baseline demographic data of study population is shown in Table 1. Average age, gender, and BMI were similar among three groups. There were significant differences in diabetic duration and HbA1C between the T2DM group without DNP and the T2DM group with DNP (all *P* < 0.01). There were significant differences in ankle brachial index, NSS (neuropathy symptom score), NDS (neuropathy disability score), VPT (vibration perception threshold), and SWM (Semmes-Weinstein monofilaments) between the control group, the T2DM group without DNP, and the T2DM group with DNP (*P* < 0.01 for ankle brachial index and NSS; *P* < 0.001 for NDS, VPT, and SWM).

**Figure 1:**
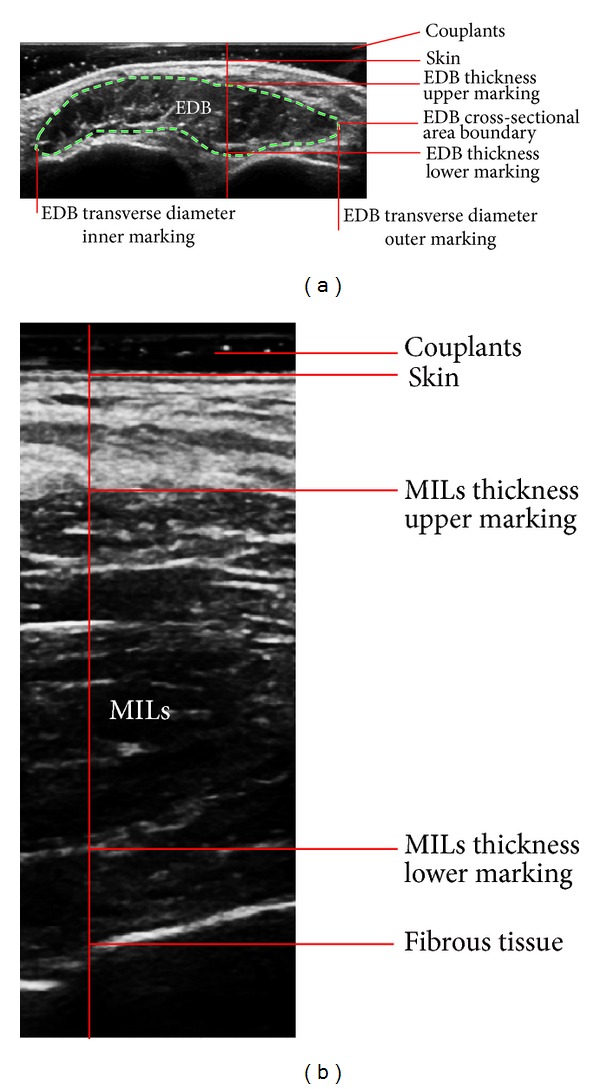
Ultrasonographic images of the extensor digitorum brevis muscle (EDB) and the muscles of the first interstitium (MILs). (a) A representative ultrasonic image of EDB, along with the enthesis (indicating lines with annotations) and boundary (dashed line) of the measured transverse diameter, thickness, and cross-sectional area. (b) A representative ultrasonic image of MILs and the enthesis (indicating lines with annotations) of the measured thickness.

**Figure 2:**
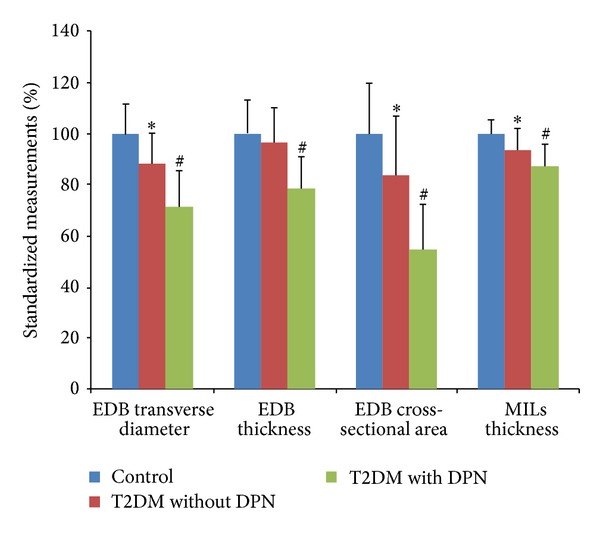
Standardized measurements of the extensor digitorum brevis muscle (EDB) and the muscles of the first interstitium (MILs). The graph shows the standardized measurements when the measured values of the control group are normalized as 100. ∗ indicates *P* < 0.01 when compared with the control group. # stands for *P* < 0.01 when compared with the T2DM without DPN group.

In the ultrasonography, the fascicles were shown as hypoechogenicity or medium echogenicity, surrounded by epimysium, fascia, and adipose tissue shown as hyperechogenicity of strips and streaks (Figure 1). The measured boundary of muscles was defined with the inner edge of fascia (see labels in Figure 1). The ultrasonographic transverse diameter, thickness, and cross-sectional area of the EDB and the thickness of MILs in patients of T2DM with DPN were significantly smaller than those in patients of T2DM without DPN (all *P* < 0.01) and those in the control subjects (all *P* < 0.01). The transverse diameter and cross-sectional area of the EDB and thickness of MILs in patients of T2DM without DPN were significantly smaller than those of the control subjects (all *P* < 0.01). Data of ultrasonographic measurement are shown in Table 2. Among the changes of parameter values in patients with DPN, the cross-sectional area of EDB decreased the most, for example, to 65.46 ± 21.65% of that in patients without DPN, while the transverse diameter of EDB decreased to 80.61 ± 16.59%, the thickness of EDB to 81.19 ± 13.02%, and the thickness of MILs to 93.50 ± 8.86% (graph in Figure 2).

The CVs were 11.9% for the transverse diameter of the EDB, 13.13% for the thickness of the EDB, 19.86% for the cross-sectional area of the EDB, and 5.62% for the thickness of the MILs.

## 4. Discussion

The present study is the first exploration of ultrasonographic detection of foot muscle atrophy in Chinese diabetic patients of T2DM with or without DPN as far as we know. The results have shown that foot muscle atrophy occurs in diabetic patients including those without DPN, which can be detected by ultrasonographic examination and measurement on EDB and MILs. The cross-sectional area of EDB may be the most sensitive parameter.

Diabetic patients have pathological changes of metabolism and microvessels along with later neuropathy, which may cause muscle atrophy. It involves complicated cellular and molecular mechanisms, including apoptosis and abnormality of ubiquitin-proteasome and lysosome systems [21, 22]. DPN especially may induce movement disorders in distal extremities such as decrease of muscle strength of foot, resulting in nonhealing ulceration and tragic amputation [23]. Thus, the foot muscle atrophy in diabetic patients is progressive, possibly early before the concurrency of DPN, which may be worsened by DPN and can cause serious consequences eventually. It is of important clinical significance to have early diagnosis of the atrophy in the patients. Practically, physical examination can find the evident muscular dystrophy and foot deformity in the patients at the late stages, but unlikely the early subtle changes [23]. Neurophysiological detection targets the electrophysiological conduction deficiency of DPN, rather than muscle atrophy. Magnetic resonance technique has excellent performance to detect the early muscle atrophy, but it is time-consuming, expensive, and inconvenient for bedside examination [24], while ultrasonography has the superiority over the above flaws.

The present study demonstrated that high-frequency ultrasonography is able to acquire clear images of foot muscle of patients with T2DM for the subsequent measurement of multiple parameters. The analysis of data shows that most measured values are smaller in the diabetic patients than those in the control subjects, and the values are even smaller in the patients with DPN than those in the patient without DPN. It suggests that high-frequency ultrasonography can detect the foot muscle atrophy not only at the late stage with DPN but also at the early stage without DPN. It also supports the early occurrence of foot muscle atrophy in diabetic patients [25].

Among the measured parameters, the cross-sectional area of EDB decreased the most. It is consistent with the fact that EDB is the earliest to shrink [26] and also implicates that EDB may serve as the best optional muscle group for the early detection of foot muscle atrophy, which is critical in the screening among large population. The early detection may provide an opportunity for the timely prevention of the foot muscle atrophy, such as optimal footwear [27, 28].

Technically, ultrasonography has been a matured application for the detection of muscle atrophy [29]. The important thing is to ensure the reproducibility of tissue measurement [30, 31]. Our study kept strict accordance with the previously described methods [13] and tested the repeatability of the measurement by analyzing CV of the parameters. Remarkably, our results have comparable reproducibility (all CVs below 4%) to that in the former study (all CVs below 6%) [13]. Larger population included in the detection of our study might also contribute to the good reproducibility.

The sensitivity of the detection may be affected by several factors. For example, the ethnic differences [32] and experiences of sports or rehabilitation training [33] may have affected the baseline of measured values in the subjects, which is critical for the following judgment of the changes. Our results suggested that the measured size of foot muscles in Chinese population may be smaller than that in Caucasians including the Danes [13]. To alleviate the risk of nondetectability, a larger population was included in our study than before [13]. This strategy successfully helped the discovery of the muscle atrophy not only in diabetic patients but also in patients without DPN. To eliminate the influence of training, individuals with such experience have been excluded after the inquiry.

Our study has limitations though. For instance, we have not coordinated the extent of possible atrophy with the specific disease course to identify the detailed relationship [12], which may help more in the early detection [23, 29]. The previous studies [12, 13] and ours used the two-dimensional images of ultrasonography for analysis. Although there is a good correlation among the thickness, cross-sectional area, and volume of muscles [34], the 3D imaging combined with the unbiased stereological analysis is expected to provide the information of volume change more directly [35].

## 5. Conclusion

Our data show that the atrophy of foot muscle in Chinese T2DM patients, especially at the early stage without DPN, can be detected by high-frequency ultrasonography. The cross-sectional area of EDB is suggested to be the most sensitive parameter, particularly for a fast screening in a large population. This convenient and sensitive detection may help timely prevention of diabetic foot and improve the prognosis.

## Figures and Tables

**Table 1:** Characteristics of the study groups.

	Control group	T2DM without DPN group	T2DM with DPN group
Number of cases	50	50	56
Age (years)	59 ± 7	59 ± 10	63 ± 7
Sex (male/female)	25/25	26/24	30/26
BMI (kg/m^2^)	24.7 ± 5.8	27.3 ± 7.1	28.1 ± 4.7
Diabetes duration (years)∗	—	6 ± 3	18 ± 5
HbA1C (%)∗	—	7.6 ± 1.9	8.8 ± 2.3
Ankle brachial index^†^	1.1 ± 0.1	1.1 ± 0.2	0.9 ± 0.2
NSS^†^	0 ± 0	2 ± 2	4 ± 2
NDS^‡^	0 ± 0	2 ± 1	12 ± 7
VPT^‡^	8 ± 2	14 ± 8	35 ± 17
SWM^‡^	3.79 ± 0.52	4.12 ± 0.63	6.55 ± 0.78

Data are means ± SD. *P < 0.01 for T2DM without DPN versus T2DM with DPN; ^†^ *P* < 0.01 for control and T2DM without DPN versus T2DM with DPN; ^‡^ *P* < 0.001 for control and T2DM without DPN versus T2DM with DPN. NSS: neuropathy symptom score; NDS: neuropathy disability score; VPT: vibration perception threshold; SWM: Semmes-Weinstein monofilaments.

**Table 2:** Measured foot muscle atrophy in Chinese diabetic patients and the matched control subjects.

	Control group (*n* = 50)	T2DM without DPN group (*n* = 50)	T2DM with DPN group (*n* = 56)
EDB transverse diameter (mm)	75.85 ± 9.03	66.93 ± 9.28^&^	53.95 ± 11.05^∗#^
EDB thickness (mm)	7.16 ± 0.94	6.91 ± 0.97	5.61 ± 0.90^∗#^
EDB cross-sectional area (mm^2^)	165.42 ± 32.86	138.10 ± 39.26^&^	90.40 ± 29.90^∗#^
MILs thickness (mm)	34.32 ± 1.93	32.16 ± 2.86^&^	30.07 ± 2.85^∗#^

∗ and & stand for comparison with the control group, *P* < 0.01; # stands for comparison with the group of T2DM without DPN, *P* < 0.01.
